# The Management of Type 2 Diabetic Patients with Hypoglycemic Agents

**DOI:** 10.5402/2012/601380

**Published:** 2012-04-04

**Authors:** G. P. Carnevale Schianca, D. Sola, L. Rossi, G. P. Fra, E. Bartoli

**Affiliations:** Dipartimento di Medicina Clinica e Sperimentale, Università del Piemonte Orientale, A. Avogadro, 28100 Novara, Italy

## Abstract

*Aims and Scope*. Aims of the paper are to suggest the best treatment to improve the glycemic control in patients with Type 2 diabetes using hypoglycemic agents, in particularly, we think that every patient is different from another one in terms of BMI, family history, duration of the disease and so on. We propose for every clinical aspect the best hypoglycemic agents to use, considering the scientific evidence and physiopathology.

## 1. Introduction

In recent years, a series of innovative molecules have been introduced to treat type 2 diabetes, a challenging task as the disease is complex and multifactorial, due to defects in insulin secretion and action, laden with a heavy impact on public health because of its growing prevalence.

A good glycemic control, the best guarantee to reduce the risk of development and/or progression of microvascular disease, remains the cornerstone of diabetes management, although its impact on macrovascular disease is still under debate. However, any strategy for glycemic control should be integrated in a context of multifactorial interventions to control all cardiovascular risk factors associated with diabetes.

In the 1990s, a patient with newly diagnosed diabetes, with a glycated hemoglobin (A_1c_) of 7.5%, systolic blood pressure of 140 mmHg and LDL-cholesterol of 130 mg/dL, would have been given the recommendation to reduce body weight and to increase physical activity. Nowadays, the same patient, in addition to life style changes, would start an intensive pharmacological treatment.

As reported by the UKPDS [1], intensive therapy, when compared to the conventional form, significantly reduced the relative risk of myocardial infarction and death from any cause. In contrast, recent clinical studies (ACCORD, ADVANCE, and VADT) [2–4] yielded surprising results: intensive care, even when lowering A_1c_ below 7%, was not associated with any significant reduction in cardiovascular (CV) mortality. Why such contradictory results? To answer this question we must analyze the diabetic patients enrolled in the above-mentioned trials: newly diagnosed patients with no prior CV event in the UKPDS, patients with long-standing disease and a high prevalence of micro- and macrovascular complication in the ACCORD, ADVANCE, and VADT trials. In a recent metaregression analysis, in addition to the duration of disease, higher body mass index (BMI) and severe hypoglycemia were found associated with a greater risk of CV events in patients undergoing intensive therapy [5].

Altogether, these data suggest that several factors can be taken into consideration when planning treatment of diabetes, including duration and stage of disease, life expectancy, risk of hypoglycemia, and risk factors for CV disease (CVD). Consequently, we must consider not only the type of pharmacological agents used to control glucose, but also the phenotype of the diabetic patient when planning modern treatment.

 Of importance to lifestyle changes and weight control, it is necessary to know the mechanism of action of all hypoglycemic agents, each endowed with specific properties. They are different from each other and should be appropriately rather than haphazardly used. The goal of treatment is not the mere reduction of hyperglycemia, possibly with each individual hypoglycemic agent, but rather to reach the best glycometabolic control while preserving *β*-cell function and quality of life.

The guidelines, proposed by the American Diabetes Association (ADA) and the European Association for the Study of Diabetes (EASD), provide a series of steps, the first of which is the use of metformin. The subsequent steps involve the progressive addition of all other hypoglycemic agents, including insulin [6].

In this article, we shall discuss when and how to use each individual hypoglycemic agent. With the exclusion of acarbose, these can be divided into two groups: those that increase the patient's secretion of insulin (sulphonylureas, glinides and incretins) and those that enhance its effectiveness (metformin and pioglitazone). The fact that hypoglycemic agents substantially have the same effect on A_1c_ does not justify their uncritical use. Their choice is not at all optional but should depend on the properties of the drug and the clinical evidence yielded by persuasive studies.

Subsequently, we shall discuss the use of these drugs in a particular group of diabetic patients, those with newly diagnosed type 2 diabetes: how should we treat them? Which drugs should we use?

## 2. When and How Should We Use Metformin?

Metformin is the only biguanide presently available almost everywhere. It acts mainly on the liver, where it decreases glucose production, thus lowering fasting plasma glucose (FPG). It may improve peripheral glucose disposal while suppressing hunger and promoting weight reduction. Since it does not increase insulin levels, although it requires the presence of endogenous insulin, metformin can also be used in patients with type 1 diabetes, when they have residual functioning pancreatic *β*-cells. Generally, it is well tolerated, the most common adverse effects being gastrointestinal. When appropriately used, the risk of lactic acidosis is minimal [7]. The UKPDS demonstrated the benefit of metformin therapy on CV outcomes, especially in overweight-obese patients [1].

These data hinge on the fact that metformin is considered “first line” oral therapy in type 2 diabetes, especially in overweight patients. An additional indication is its low cost and the fact that it can be associated with every other glucose-lowering agent, including insulin.

Metformin should be taken with, or immediately after, a meal. To avoid its common gastrointestinal effect, it should be introduced using initial low doses, gradually titrated upward. People should be informed that these adverse effects often improve after a few days of continued treatment. Metformin should be discontinued during severe illnesses like myocardial infarction, pneumonia, severe infection, and/or dehydration, as it may aggravate tissue hypoxia and accumulate when renal function is impaired. It may be appropriate, in these cases, to use other glucose-lowering agents, including insulin. It is a good rule, given the increasingly wide use of iodine contrast agents even when serum creatinine is increased, to discontinue metformin prior to these radiographic procedures.

## 3. When and How Should We Use Sulphonylureas/Glinides?

Sulphonylureas increase the release of endogenous insulin from *β*-cell. They exert effects on A_1c_ similar to those of metformin, but their use entails a greater risk of hypoglycemia and of undesired weight gain. Even though this risk is lower with the newer sulphonylureas (glipizide, glimepiride) [8], these episodes, more frequent and dangerous in the elderly, severely limit their use. In addition, an unwanted weight gain of approximately 2 kg is common after introducing sulphonylureas.

Old age, renal impairment, and liver disease are conditions where sulphonylureas should not be used.

In patients suffering from inadequate glycemic control, sulphonylureas can achieve significant improvements when added to metformin [9]. Another controversial aspect of sulphonylureas treatment is their CV safety. In comparison with metformin, sulphonylureas treatment seems associated with a significant increase in adverse CV outcomes [1, 10, 11]. Although still under debate, this issue must be kept in mind when planning long-term treatment.

Like sulphonylureas, glinides stimulate insulin secretion. Their shorter half-life requires a more frequent administration. Like sulphonylureas, glinides cause a similar weight gain, although hypoglycemia seems less frequent [12].

Even though sulphonylureas are the most widely used oral antidiabetic agents, in the future they will be used more sparingly. In our opinion, this scenario is inevitable, considering the pathophysiology of type 2 diabetes. Patients with type 2 diabetes very often have lost 50% of their *β*-cell function at the time of diagnosis [13]. Consequently, sulphonylureas, while causing a deceptive improvement of A_1c_, do in fact accelerate *β*-cell dysfunction by imposing an additional burden on residual *β*-cells secretion. The clinical correlate of this phenomenon is known as “secondary failure” and it represents the inevitable fate of all oral hypoglycemic agent, especially sulphonylureas. We believe that the less we use of sulphonylureas, the less will be the relevance of this “secondary failure.” Given that *β*-cell dysfunction is prominent at the time of diagnosis of diabetes and given the rate of decline of *β*-cell function with time [10, 13], it does not seem necessary to trigger the production of endogenous insulin by a dysfunctional *β*-cell, when the plasma levels of insulin itself are already, in almost all instances, elevated. Given this evidence, drugs enhancing the efficacy of insulin would appear more appropriate.

In our opinion, because of the aforementioned considerations, sulphonylureas should no longer be used.

## 4. When and How Should We Use *α*-Glucosidase Inhibitors?

The inhibition of intestinal *α*-glucosidase in the brush border of the small intestine delays the absorption and digestion of complex carbohydrates. Although these agents do not increase the response to insulin of peripheral tissues, their ability to curb the rise in plasma glucose after meals can reduce plasma insulin levels and the need to administer insulin. As predicted by their mechanism of action, hypoglycemic adverse effects and weight gain do not occur [14]. There are no data on long-term effects of *α*-glucosidase inhibitors in terms of mortality, morbidity, and quality of life. However, we believe that the significant effect in preventing the conversion to type 2 diabetes in patients with impaired glucose tolerance (IGT) exerted by acarbose, the *α*-glucosidase inhibitor most widely used, compared with placebo [15], highlights the possible clinical benefits of acarbose. While these benefits are not established yet, they will be in all likelihood demonstrated in the near future.

The patients best suited for *α*-glucosidase inhibitor treatment are those with high or excessive postprandial glucose levels. This specificity expands their use to all patients, including type 1 diabetes.

These agents are quite safe, but they often cause dose related gastrointestinal adverse effects like disturbing bloating, flatulence and diarrhea. These symptoms, the prevalence of which is similar to that observed with metformin [16], mainly arise from the fermentation of undigested carbohydrates by colonic bacteria.

The *α*-glucosidase inhibitors should be introduced in low doses, with a gradual step-wise escalation. Some patients do not tolerate the higher doses, in which case dose reduction is appropriate.

We believe that *α*-glucosidase inhibitors, if tolerated, can be used alone or in combination with other agents for treating all patients with type 2 diabetes.

## 5. When and How Should We Use Thiazolidinediones?

Thiazolidinediones increase whole-body insulin sensitivity by activating nuclear receptors. Presently, only pioglitazone is available for patients' use. In this report, we shall not mention rosiglitazone, which is not licensed for use yet, and in few countries it has recently been taken off the market because of undesired CV side-effects.

The availability of an oral drug that can effectively counter insulin resistance, a crucial pathophysiological component of diabetes [17], has expanded our treatment options. However, it is essential to use the drug properly and the physicians who really know its properties will not resort to its unselected use.

Pioglitazone is effective in lowering A_1c_ when used alone as well as in combination with metformin, sulfonylurea or insulin [18]. Weight gain, fluid retention, and risk of fractures (only in women) are its main side effects [16, 19]. It seems that fat accumulation is largely subcutaneous, with redistribution of fat from visceral deposits, while fluid retention usually appears as peripheral edema, with a consequent risk of new or worsening congestive heart failure (CHF) in predisposed individuals [6].

After the evidence reporting the unwanted side effects of the other thiazolidinediones, the CV safety of pioglitazone is currently under debate. A Cochrane systematic review reported insufficient evidence to draw conclusions on CV and non-CV outcomes [20].

Substantially pioglitazone, compared with treatments based on different drugs, significantly reduced composite CV outcomes [21, 22], even when used in diabetic patients with a long disease duration and previous myocardial infarction or stroke [18]. On the other hand, the higher risk of CHF reported should not be disregarded [21, 23].

In our opinion, pioglitazone is an important option for diabetes treatment. There is concern stemming from the fact that the drug acts on nuclear receptors of all cells, raising questions on its possible long-term effects. Recently, an increased incidence of bladder cancer attributed to pioglitazone use has been reported [24]. However, the benefits of pioglitazone may outweigh the associated risks. Only patients with marked insulin resistance should be selected to receive the drug. We believe that the association of pioglitazone with metformin is very advantageous and that it should be encouraged also for the beneficial effects on the atherogenic lipid profile induced by pioglitazone itself [25]. When ankle edema occurs, it is usually appropriate to discontinue the drug.

Pioglitazone use has been recently extended to nonalcoholic fatty liver disease and prediabetes [26, 27]. It is a drug with an important clinical potential, its strength being its attractive mechanism of action in countering insulin resistance.

## 6. When and How Should We Use Incretins?

The observation that insulin is released more efficiently after an oral glucose load than with an intravenous injection is known as the incretin effect [28]. This effect, occurring when intestinally derived peptides, as the glucagon-like polypeptide 1 (GLP-1), stimulates insulin release in response to oral glucose and is rapidly lowered by the enzymatic digestion of the peptides operated by glycoprotein dipeptidyl peptidase (DPP-4). The incretins work by enhancing the sensitivity of *β*-cell to glucose, which causes enhanced glucose-dependent insulin release. Substantially, they exert an insulinotropic action secondary to a marked suppression of *α*-cells; thus, they protect the *β*-cell from an unfavorable environment where glucagon is prominent [29], making available the functioning of *β*-cell that was hampered by *α*-cell overactivity.

It is possible to enhance the incretin pathway in two ways: by slowing the peptide breakdown through inhibition of DPP-4 release, or by enhancing the action of GLP-1 itself.

The DPP-4 inhibitors, now available in clinical practice, prolong the action of endogenous GLP-1. These oral drugs, similarly to other hypoglycemic agents, were shown to be effective in lowering A_1c_, [30]. They are well tolerated, weight neutral, and they do not induce hypoglycemia [31].

GLP-1 mimetics, administered only subcutaneously, are resistant to the breakdown by the DPP-4 enzyme, resulting in more prolonged action. They exert systemic actions compared to DPP-4. In addition to the insulinotropic action in response to ingested glucose and to the inhibition of inappropriate glucagon secretion, they delay gastric emptying, resulting in slower absorption of glucose following meals, while promoting satiety attended by significant weight reduction [29].

The current available GLP-1 mimetics are exenatide, which has a half-life of four hours requiring a twice daily subcutaneous injection, and liraglutide, which has a half-life of approximately 12 hours, requiring once daily subcutaneous injection. The reduction of A_1c_ is well documented, similar to that afforded by insulin [31] and more important than that obtained with other glucose-lowering agents [31, 32].

Both exenatide and liraglutide are generally well tolerated, while severe hypoglycemia is rare. The most common adverse events are gastrointestinal, especially nausea. Data purporting to show an increase incidence of acute pancreatitis in patients treated with incretins is considered inconclusive [31, 32].

Our opinion is that a large proportion of people with type 2 diabetes can experience an important advantage from incretin therapy. The beneficial action on glucagon metabolism implemented by incretin is unique. It would not be wise to disregard this advantage, since in diabetes increased glucagon levels are invariably present and constitute an important pathophysiologic component of the disease. Actually, incretins may be used to improve glycemic control in obese adults for whom weight loss is a priority, although we believe that these drugs will be more effective if used early in the course of the disease, when the *β*-cell can still regain part of its function.

## 7. Pharmacological Management of Individuals Newly Diagnosed with Type 2 Diabetes

Most diabetic patients are identified later in the course of disease, when the FPG is much higher than 140 mg/dL, and A_1c_ concentration is well above 7–7.5%.

This clinical situation is not ideal for two relevant reasons.

The first one is the clinical inertia. This, representing the failure to initiate or advance therapy in a patient who is not at the evidence-based therapeutic goal, is now considered a primary reason for poor metabolic control [33]. The causes of clinical inertia identified are multifactorial, including the attitude of physicians, organizational aspects, and compliance of patients. The strongest barriers raised against a more advanced therapeutic intervention or intensification treatment are the risk of hypoglycemia and of weight gain. Therefore, we believe that the early use of the new glucose-lowering agents, like incretins, can minimize these risks with a positive impact on disease progression and a delay in the need of insulin administration.

The second reason is that most patients with type 2 diabetes, as well demonstrated in UKPDS [10], have already lost 50% of their *β*-cell function at the time of diagnosis, underscoring the important role of *β*-cell dysfunction, in addition to that of insulin resistance. In the same study, there was evidence of a relentless progressive loss of *β*-cell function over the ensuing years, associated with glycemic deterioration, which occurred regardless of treatment. At the time of patient enrollment, beside life style intervention, only insulin, sulphonylureas, and metformin were used. We do not know whether the use of pioglitazone and incretins could have better preserved *β*-cell function. As previously discussed, both pioglitazone and incretins seem to be able to spare secretion. In fact, pioglitazone can slow down the progression to type 2 diabetes when used in IGT patient [27], while both GLP-1 mimetics and DPP-4 inhibitors improve *β*-cell function measured by HOMA-*β* [30].

These data, taken together, suggest that, if it is true that the earlier the treatment is begun the better is its outcome, it is also true that the earlier we diagnose diabetes, the more effectively we can control it.

Recently, the ADDITION-Europe study compared intensive multifactorial therapy with routine care in type 2 diabetic patients detected by screening [34]. Unlike other clinical trials, as ACCORD, ADVANCE, and VADT [2–4], the ADDITION-Europe study enrolled diabetic patients whose diagnosis had been reached by a programmed screening design. Although only a small trend towards CV benefit (−17%) was recorded from intensive treatment compared with routine care, this study conveys an important message. In ACCORD, ADVANCE, and VADT trials, where the diabetic patients had a long duration of disease and/or previous CV events, the intensive multifactorial therapy, compared with the conventional one, was associated to a significantly increased incidence of CV events. This suggests that any treatment is more effective when started early. In the case of diabetes, any treatment introduced earlier than the time of routine diagnosis will turn out to be advantageous. For example, the screening of diabetes by the oral glucose tolerance test (OGTT) can also dictate the choice of a specific drug. We recently reported evidence of a heterogeneous presentation of type 2 diabetes diagnosed by the OGTT [35]. Based on the OGTT, 103 subjects out of 1277 (8.1%) were found affected by new onset type 2 diabetes. On the basis of the glucose cut-off values for diagnosing type 2 diabetes, FPG > 125 and/or 2 hour plasma glucose (2hPG) ≥ 200 mg/dL, three different disease presentations are possible. In fact, out of 103 newly diagnosed type 2 diabetics, 22 were diabetics only because of their elevated FPG, 55 only because of their elevated 2hPG, and, finally, 26 because of both. These results are consistent with a heterogeneous presentation of type 2 diabetes, detectable before the common routine diagnosis, thus allowing the possibility of specific treatments. When FPG is elevated, probably metformin and pioglitazone are more suitable [6, 17], while pioglitazone, acarbose, and incretins are more suitable when 2hPG is elevated [15, 17, 30].

## 8. Conclusion

Type 2 diabetes is in significant expansion. Treatment is often conservative and limited to an unsatisfactory control focused mainly on glucose values. The common therapeutic scenario relies on the use of an oral agent followed by the step addition of others delaying insulin treatment.

Presently, innovative molecules have been made available: physicians need to understand them properly. We strongly recommend that diabetes be treated as early as possible. Only in this way, the catastrophic consequences of the disease can be effectively prevented and limited. 
